# Dementia Risk in Older Veterans With Frailty: A Cross-Sectional Study

**DOI:** 10.1093/geroni/igab046.3477

**Published:** 2021-12-17

**Authors:** Christian Gomez Hernandez, Alma Diaz, Ahmadou Sow, Gauty Athouriste, Ezekiel Ijaopo, Jorge Ruiz

**Affiliations:** 1 Miami VA Healthcare System, Miami, Florida, United States; 2 Miami VAMC, Miami, Florida, United States; 3 University of Miami/Jackson Health System, Miami, Florida, United States; 4 VAMC, Miami, Florida, United States

## Abstract

Frailty, a clinical syndrome characterized by vulnerability to stressors resulting from multisystemic loss of physiological reserve, predicts future cognitive decline. However, frailty has also been proposed as a dementia risk factor, predicting future cognitive impairment. The study aim was to determine frailty in older veterans and its association with risk of dementia. Community-dwelling Veterans ≥50 years completed a mailed socio-demographic questionnaire and Self-Administered Gerocognitive Examination (SAGE), July 2019-May 2020. The information was complemented with EHR data. We calculated the CAIDE score, a validated tool predicting dementia (≥6 points= high risk 20 years later) and the 31-item VA frailty index data (frail ≥.20, non-frail ≤.20). After adjusting for socio-demographic characteristics, smoking, alcohol/substance abuse, OSA and anticholinergic use, odds ratio (OR) and 95% CI were calculated using BLR to assess the cross-sectional association between frailty and dementia risk (CAIDE ≥6 points and MCI). The survey response rate was 19.75% (1,073 of 5,432). Participants mean age was 68.38 (SD=8.49) years, 57.50% (n=617) Caucasian, 69.34% (n=744) non-Hispanic, 95.81% (n=1,028) male, and 36.72% (n=394) frail. 11.84%(n=127) screened positive for MCI and 15.38% (n=165) for dementia. 689 (75.88%) veterans were at high risk for dementia of whom 426 (61.83%) were non-frail and 263 (38.17%) were frail. Frailty was cross-sectionally associated with higher risk for dementia in older Veterans, adjusted OR:1.45 (95%CI:1.016-2.070), p=.041. The mailed screening was a feasible and practical approach to screen for dementia risk. Early identification of patients with frailty can help in the implementation of interventions aimed at preventing or delaying dementia.

